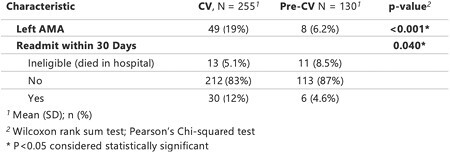# 744 The Unhoused Burn Population: An Alarming Increase of Leaving Against Medical Advice and Unplanned Readmissions

**DOI:** 10.1093/jbcr/irae036.287

**Published:** 2024-04-17

**Authors:** Noah Speiser, Sean J Donohue, Trevor A Pickering, Christopher H Pham, Maxwell B Johnson, Justin Gillenwater, Haig A Yenikomshian

**Affiliations:** Keck School of Medicine, University of Southern California, Rolling Hills, CA; University of Southern California, Studio City, CA; University of Southern California, Los Angeles, CA; Keck Medicine of USC, Los Angeles, CA; Keck School of Medicine, University of Southern California, Rolling Hills, CA; University of Southern California, Studio City, CA; University of Southern California, Los Angeles, CA; Keck Medicine of USC, Los Angeles, CA; Keck School of Medicine, University of Southern California, Rolling Hills, CA; University of Southern California, Studio City, CA; University of Southern California, Los Angeles, CA; Keck Medicine of USC, Los Angeles, CA; Keck School of Medicine, University of Southern California, Rolling Hills, CA; University of Southern California, Studio City, CA; University of Southern California, Los Angeles, CA; Keck Medicine of USC, Los Angeles, CA; Keck School of Medicine, University of Southern California, Rolling Hills, CA; University of Southern California, Studio City, CA; University of Southern California, Los Angeles, CA; Keck Medicine of USC, Los Angeles, CA; Keck School of Medicine, University of Southern California, Rolling Hills, CA; University of Southern California, Studio City, CA; University of Southern California, Los Angeles, CA; Keck Medicine of USC, Los Angeles, CA; Keck School of Medicine, University of Southern California, Rolling Hills, CA; University of Southern California, Studio City, CA; University of Southern California, Los Angeles, CA; Keck Medicine of USC, Los Angeles, CA

## Abstract

**Introduction:**

Unhoused burn patients (UBP) have historically been more likely to leave against medical advice (AMA) and suffer worse health outcomes compared to the general population. The COVID-19 (CV) pandemic has created major strain on the healthcare system that has resulted in worse overall health outcomes in burn patients. Currently, there is sparse data on how UBP have fared since the start of CV. We sought to investigate how CV impacted treatment for UBP, specifically the rate of leaving AMA and unplanned readmissions.

**Methods:**

We conducted a retrospective chart analysis of UBP admitted to a regional burn center from 2014 through 2022. March 2020 was used as the cutoff to separate the cohorts into pre-CV and CV patients. Outcomes studied included leaving AMA and readmission rates within 30 days. Data analysis included distributions of continuous variables using the Wilcoxon rank sum test and dichotomous variables using Pearson’s Chi-squared test.

**Results:**

385 patients met inclusion criteria, of which 130 were placed in the pre-CV and 255 were placed in the CV group. UBP were significantly more likely to leave AMA during CV (19.2% vs. 6.2%, p< 0.001). These patients also experienced a significantly higher 30-day readmission rate during CV (11.8% vs. 4.6%, p< 0.05).

**Conclusions:**

CV has changed the paradigm of many health systems and the approach to treating UBP is no exception. Our findings identify two alarming increases: the rate of leaving AMA and the readmission rate in this population. While the root of this is unclear, these factors represent the inadequate care being provided to this vulnerable patient population. Future research should identify the root causes of these factors and identify early interventions to mitigate them.

**Applicability of Research to Practice:**

Leaving AMA is associated with worse health outcomes and understanding the driving force behind this increase can improve care for this population.